# Influence of target dose heterogeneity on dose sparing of normal tissue in peripheral lung tumor stereotactic body radiation therapy

**DOI:** 10.1186/s13014-021-01891-6

**Published:** 2021-08-30

**Authors:** Zhigong Wei, Xingchen Peng, Yan Wang, Lianlian Yang, Ling He, Zheran Liu, Jingjing Wang, Xiaoli Mu, Ruidan Li, Jianghong Xiao

**Affiliations:** 1 Department of Biotherapy, Cancer Center, West China Hospital, Sichuan University, Chengdu, Sichuan China; 2 School of Computer Science, Sichuan University, Chengdu, 610000 China; 3 Department of Radiation Oncology, Cancer Center, West China Hospital, Sichuan University, No.37 Guoxue Alley, Wuhou District, Chengdu, 610041 Sichuan China

**Keywords:** Dose heterogeneity, Dose sparing, Stereotactic body radiation therapy, Volumetric-modulated arc therapy

## Abstract

**Objective:**

To evaluate the influence of target dose heterogeneity on normal tissue dose sparing for peripheral lung tumor stereotactic body radiation therapy (SBRT).

**Methods:**

Based on the volumetric-modulated arc therapy (VMAT) technique, three SBRT plans with homogeneous, moderate heterogeneous, and heterogeneous (HO, MHE, and HE) target doses were compared in 30 peripheral lung tumor patients. The prescription dose was 48 Gy in 4 fractions. Ten rings outside the PTV were created to limit normal tissue dosage and evaluate dose falloff.

**Results:**

When MHE and HE plans were compared to HO plans, the conformity index of the PTV was increased by approximately 0.08. The median mean lung dose (*MLD*), *V*_*5*_, *V*_*10*_, *V*_*20*_ of whole lung, *D*_*2%*_, *D*_*1cc*_, *D*_*2cc*_ of the rib, *V*_*30*_ of the rib, *D*_*2%*_ and the maximum dose (*D*_*max*_) of the skin, and *D*_*2%*_ and *D*_*max*_ of most mediastinal organs at risk (OARs) and spinal cord were reduced by up to 4.51 Gy or 2.8%. Analogously, the median *D*_*max*_, *D*_*2%*_ and mean dose of rings were reduced by 0.71 to 8.46 Gy; and the median *R*_*50%*_ and *D*_*2cm*_ were reduced by 2.1 to 2.3 and 7.4% to 8.0%, respectively. Between MHE and HE plans there was little to no difference in OARs dose and dose falloff beyond the target. Furthermore, the dose sparing of rib *V*_*30*_ and the mean dose of rings were negatively correlated with the rib and rings distance from tumor, respectively.

**Conclusions:**

For peripheral lung tumor SBRT, target conformity, normal tissue dose, and dose falloff around the target could be improved by loosening or abandoning homogeneity. While there was negligible further dose benefit for the maximum target dose above 125% of the prescription, dose sparing of normal tissue derived from a heterogeneous target decreased as the distance from the tumor increased.

**Supplementary Information:**

The online version contains supplementary material available at 10.1186/s13014-021-01891-6.

## Background

Worldwide, lung cancer remains the leading cause of cancer incidence and mortality [1]. Also, the lung is a common site of metastases for various other cancers. Radiotherapy is an alternative treatment method for inoperable patients. Yet, local control of conventional fraction radiotherapy has historically been poor due to insufficient total radiation doses [2]. Stereotactic body radiation therapy (SBRT) can very precisely deliver a high dose of radiation to an extracranial target, using either a single dose or a small number of fractions [3]. The clinical benefits of SBRT have been demonstrated by several prospective studies for both early-stage, non-small cell lung cancer (NSCLC) and pulmonary oligo-metastases [4–6].

Meanwhile, a high dose per fractional treatment is more likely to cause late effects, which can be very destructive and result in significant dysfunction to the treated tissues. It can be devastating to quality of life and even deadly [7]. Some lung SBRT studies indicate that toxicities in different organs at risk (OARs), including lung [8], chest wall [9–11], skin [12], esophagus [13], great vessels [14], trachea, and bronchus [15], are significantly correlated with the radiation dose of corresponding OARs [8, 16]. Therefore, it is of great importance to reduce radiation dose to normal tissues in SBRT. Specialized treatment planning is required to deliver the dose with as high as possible a target conformity and steep dose gradients beyond the target [17].

Because heterogeneous radiotherapy plans allow for an equal or lower dose of OARs compared to homogeneous plans [18, 19], clinicians prefer to loosen or abandon homogeneity of the target dose with SBRT. Hot spots within target volumes are generally viewed to be clinically desirable, as long as there is no spillage into normal tissues [20]. However, there is still no consensus on the dose heterogeneity level inside the target. RTOG recommends a selection of the prescription isodose surface set to a value between 60 and 90% of the dose at the center of the planning target volume (PTV) or the maximum dose (*D*_*max*_) [21]. Therefore, there is a lot of variation in target heterogeneity for SBRT plans, and which level of dose heterogeneity is the most optimal for SBRT in lung tumor remains unclear. Furthermore, the quality of a radiotherapy plan is typically dependent on the experience and preference of its planners [22–25]. Consequently, previous dosimetry studies of SBRT plans are almost all based on manual planning [23–26], which makes it difficult to guarantee unbiased comparison. In the present study, we developed an automatic planning program for SBRT to reduce reliance on the experience and preference of planners and to evaluate the relationship between dose sparing of normal tissue with different levels of dose heterogeneity.

## Methods

### Patient eligibility

A total of 30 patients, who were treated with SBRT at West China Hospital between April 2011 and March 2017 for peripheral lung cancer or pulmonary oligo-metastases, were enrolled. The characteristics of the patients are shown in Table 1.

**Table 1:** The characteristics of patients

Variable	*N*	%
Age (years)		
Median (range)	53 (36–80)	
Gender		
Male	23	76.7
Female	7	23.3
Histology		
Primary	15	50
Metastatic	15	50
Site		
Right	13	43.3
Left	17	56.7
PTV volume (cm^3^)		
Median (range)	18.21 (9.48–29)	

PTV, planning target volume

### Treatment planning

Each patient was immobilized in a stereotactic body frame (SBF, Elekta Oncology System, Sweden) in the supine position with arms raised above the head. The intravenous contrast-enhanced 4D-CT (SOMATOM Definition AS+, SIEMENS, 120 kVp, 90 mAs) covering the total lung volume was obtained. All the CTs were reconstructed in 3 mm slice thickness and transferred to the RayStation treatment planning system (TPS) (RaySearch Laboratories, v4.7). The details of localization, simulation, immobilization, delineation of target and OARs volumes, and prescription dose constraints were discussed in RTOG 0915 [21].

An automatic SBRT planning software for automatically creating the planning auxiliary structures, adding beams and initial objectives and constraints, adjusting parameters and optimization was developed based on the RayStation TPS. The program simulated the process of manual plan design, and included adding plan objectives and constraints, adjusting the parameters in the process of optimization. Objectives and constraints were set according to the prescription. Parameter adjustment was based on each optimized objective value, to ensure the objective value was in the range of 10 to 30 times of tolerance (tolerance = 0.0001). The total number of iterations per patient was arbitrarily set to 10. The minimum precision of automatic adjustment was 2 cGy, which is far more precise than that of manual adjustment (more than 50 cGy in most cases). Using the same initial planning conditions for one randomly selected patient, 10 automated and 10 manual SBRT plans were generated to assess the repeatability. Variation coefficient was calculated as follows: standard deviation/mean value × 100%. Greater than 2% was considered to be poor repeatability.

The prescription dose to PTV was 48 Gy in 4 fractions. Based on the average CT of each patient, three SBRT plans with homogeneous, moderate heterogeneous, and heterogeneous target doses (the HO, MHE, and HE plans) were generated and customized to the accelerator (Elekta Versa HD, Elekta Oncology, UK) with a 6-MV photon beam, in which the PTV dose was controlled in 90–110%, 90–125% and 90–∞% of the prescription dose, respectively. VMAT technique was used for all plans, while the collapsed cone algorithm was performed to compute the final dose. The *D*_*max*_ in the HO and MHE plans were based on the selection of the prescription isodose surface set to 90% and 80% of *D*_*max*_, respectively [21]. Given the uncertainty of the minimum dose (*D*_*min*_), at least *D*_*99%*_ (*D*_*V*_ is the absorbed dose that covers a specified volume *V*) must be ≥ 90% of the prescription dose. Two coplanar full arcs, the same initial objectives and constraints, the collapsed cone algorithm, and a 2 mm grid were used. Ten rings (5 mm width for each ring) outside the PTV were created to limit the dose of normal tissue and evaluate dose falloff.

### Plan analysis

All automatic plans were independently assessed by three radiation oncologists using a previously described standard [27], including that target doses meet prescription goals and doses of OARs reach or are below the dose-volume limits. Using a 3-point scale, the best target coverage or OAR sparing would score 3 points and the worst would score 1 point. The overall score for each plan was the weighted summation of all the scores (33% for the weights of target coverage and 67% for the weights of OAR sparing). The mean value of three overall scores by three oncologists for each plan was regarded as each plan’s final score.

The Heterogeneity Index (*HI, HI* = (*D*_*2%*_*-D*_*98%*_)/*D*_*50%*_) was calculated according to ICRU report 83 [28], and the Conformity Index (*CI, CI* = (*TV*_*PIV*_ × *TV*_*PIV*_)/(*TV* × *PIV*) [N.B. *TV*_*PIV*_ = PTV volume within the prescription isodose volume, *TV* = PTV volume, and *PIV* = prescription isodose volume]) was calculated according to the Paddick Index equation [29]. The tumor to OAR distance (defined as the minimum distance from the tumor edge to OAR edge) was measured. Differences were analyzed by the Friedman test (among the three plans) or Wilcoxon signed ranks test (between two plans), and *P* < 0.05 (2-tailed, Friedman) and *P* < 0.017 (α/3, 2-tailed, Wilcoxon) were considered to be statistically significant. The correlation between two variables was analyzed by Spearman's rank correlation, and *P* < 0.05 (2-tailed) was considered to be statistically significant.

## Results

All of the 10 automatic plans generated for repeatability evaluation had the same dose distribution, including the dose of PTV and OARs and dose falloff (all variation coefficients < 2%). On the contrary, repeatability of the manual plans varied greatly. Variation coefficients for many parameters were greater than 5%, and those of mediastinal OARs were even more than 10%.

According to the evaluation of three radiation oncologists, MHE plans achieved the highest final scores regarding target coverage and OAR sparing (median, 15.83; range, 13.89–16.78), which was superior than HO plans (median, 14.56; range, 12.67–15.67; *P* < 0.001) and HE plans (median, 15.05; range, 13.56–16.11; *P* = 0.001) (Additional file 1: Figure 1).

**Fig. 1:**
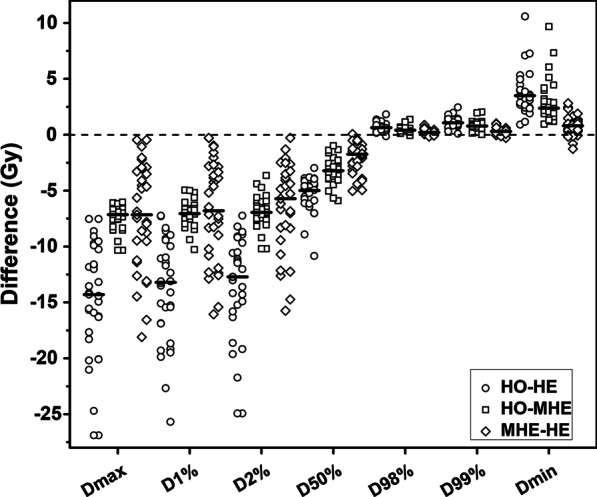
Pairwise comparisons of PTV dose. Each data point was derived from subtracting the value achieved with one plan from the one achieved with another plan. Horizontal bars indicate median values. PTV = planning target volume; *D*_*max*_ = maximum dose; *D*_*min*_ = minimum dose; *D*_*V*_ = absorbed dose that covers a specified fractional volume *V*; HO, MHE, and HE plans = homogeneous, moderate heterogeneous, and heterogeneous plans

**Fig. 2:**
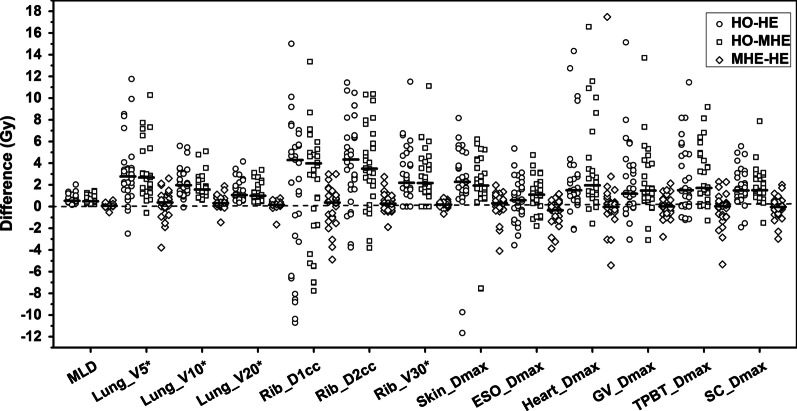
Pairwise comparisons of OARs doses. Each data point was derived from subtracting the value achieved with one plan from the one achieved with another plan. Horizontal bars indicate median values. OARs = organs at risk; TPBT = trachea and proximal bronchial tree; *MLD* = mean lung dose; ESO = esophagus; GV = great vessels; SC = spinal cord; *D*_*max*_ = maximum dose; *D*_*1cc*_ = minimum absorbed dose that covers 1 cc of the volume; *D*_*2cc*_ = minimum absorbed dose that covers 2 cc of the volume; *V*_*D*_ = volume that receives at least the absorbed dose *D* Gy; HO, MHE, and HE plans = homogeneous, moderate heterogeneous, and heterogeneous plans. *percentage

**Fig. 3:**
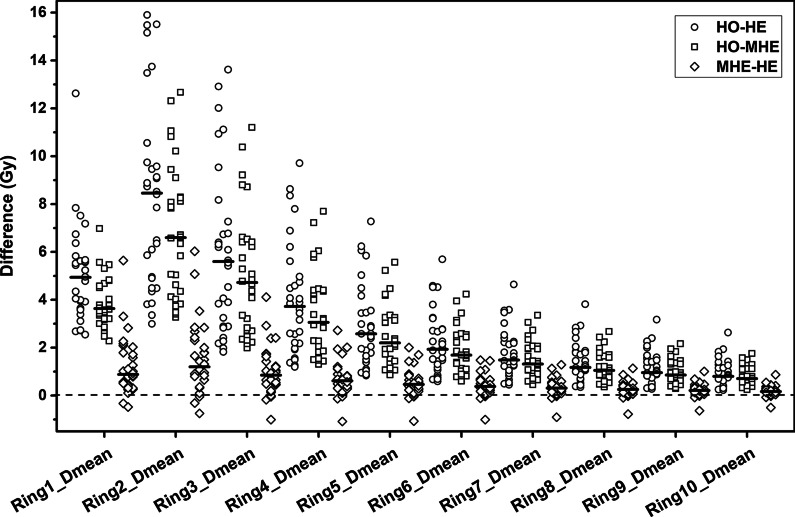
Pairwise comparisons of Rings dose. Each data point was derived from subtracting the value achieved with one plan from the one achieved with another plan. Horizontal bars indicate median values. *D*_*mean*_ = mean dose; HO, MHE, and HE plans = homogeneous, moderate heterogeneous, and heterogeneous plans

Figures 1, 2 and 3 present the dose differences of PTV, OARs, and the rings for the three kinds of plans. The statistical dose comparisons are shown in the Additional file 2: Tables 1–3. The number of patients exceeding the dose threshold are shown in Table 2. All dosimetric parameters met the prescription dose constraints except *R*_*50%*_ and *R*_*100%*_ (ratio of 50% and 100% prescription isodose volume to the PTV volume). The monitor units (MUs) of the three plan types were as follows (by order of magnitude): HO plans < MHE plans < HE plans. The maximum difference of MUs was found between HO and HE plans, where the median increase was 400 (range, 170–895) MUs.

**Table 2:** Number of patients exceeding the dose threshold

	*MLD*		Lung *V*_*20*_			Rib *D*_*2cc*_	*R*_*50%*_	*R*_*100%*_
	> 4 Gy	> 6 Gy	> 4%	> 10%	> 12%	> 30.8 Gy	> Threshold of RTOG 0915	> Threshold of RTOG 0915
HO plans	12	5	13	3	1	19	22	3
MHE plans	9	1	10	1	0	16	11	0
HE plans	9	1	9	1	0	15	6	0

*MLD*, mean lung dose; *V*_*20*_, volume that receives above 20 Gy; *D*_*2cc*_, minimum absorbed dose that covers 2 cc of the volume; *R*_*50%*_ and *R*_*100%*_, ratio of 50% and 100% prescription isodose volume to the PTV volume; HO, MHE and HE plans, homogeneous, moderate heterogeneous and heterogeneous plans

### PTV dose

*D*_*50%*_ and *HIs* of three kinds of plans, by order of magnitude, were as follows: HO plans < MHE plans < HE plans. For *CIs*, the values calculated from both MHE and HE plans were greater than those calculated from HO plans, where the median differences were approximately 0.080. There was no statistical difference in *CIs* between MHE and HE plans (*P* = 0.165).

### OARs dose

For most OARs, the doses in MHE and HE plans were lower than those in HO plans, where the median doses were reduced by up to 4.51 Gy or 2.8%. On the contrary, for dose parameters of OARs between MHE and HE plans, the differences existed only in *V*_*10*_, *V*_*20*_, *MLD* of the whole lung, *D*_*2%*_, *D*_*2cc*_ and *V*_*30*_ of the rib, *D*_*2%*_ of the skin, and *D*_*2%*_ of the esophagus, where the differences were less than 0.26 Gy or 0.3%.

#### Lung

The *MLD* and *V*_*20*_ of the whole lung in MHE and HE plans were lower than those in HO plans, where the median dose reduction was approximately 0.50 Gy (range, 0.16–2.03 Gy) and 1% (0.3–4.2%), respectively. Also, the numbers of patients whose *MLD* exceeded 6 and 4 Gy and *V*_*20*_ exceeded 12%, 10% and 4% in MHE and HE plans were less than those in HO plans. Moreover, the *V*_*5*_ and *V*_*10*_ of the whole lung in MHE and HE plans were lower than those in HO plans, where the median dose reduction ranged from 1.6 to 2.8%.

#### OARs of chest wall

For the rib, the median *D*_*2%*_, *D*_*2cc*_ and *D*_*1cc*_ were reduced by 3.85, 3.50 and 3.99 Gy (MHE plans *vs.* HO plans), and 4.51, 4.34 and 4.29 Gy (HE plans *vs.* HO plans). The median reductions of the rib *V*_*30*_ in MHE and HE plans were both 2.2% compared to HO plans. Furthermore, the dose sparing of *V*_*30*_ was negatively correlated with the tumor to rib distance (*ρ* = − 0.685, *P* < 0.001, MHE plans and HO plans; *ρ* = − 0.680, *P* < 0.001, HE plans and HO plans). For skin, the median *D*_*max*_ in MHE and HE plans were reduced by 1.94 and 2.28 Gy, respectively, compared to HO plans.

#### Mediastinal OARs and spinal cord

For most mediastinal OARs including esophagus, heart, great vessels, trachea, and proximal bronchial tree (TPBT), the doses in MHE and HE plans were lower than those in HO plans, where the median dose improvements were 0.87–1.95 Gy. Similarly, the median *D*_*max*_ of the spinal cord in MHE and HE plans was reduced by approximately 1.50 Gy compared to those in HO plans.

### Dose falloff

The median *R*_*50%*_ reductions were 2.1 (range, 0.6–10.2, *P* < 0.001, MHE plans *vs*. HO plans) and 2.3 (0.4–13.1, *P* < 0.001, HE plans *vs*. HO plans). For HE plans, only 6 patients with a PTV volume of less than 7.5 cc did not meet the *R*_*50%*_ requirements of RTOG 0915, while for MHE and HO plans these patient numbers were 11 and 22, respectively. The *D*_*max*_ (% of dose prescribed) 2 cm from the PTV in any direction (*D*_*2cm*_) for MHE and HE plans was lower than those for HO plans, where the median *D*_*2cm*_ reductions were 7.4% for MHE plans *vs*. HO plans (0.3% to 21.9%) and 8.0% for HE plans *vs*. HO plans (− 0.4% to 22.6%). However, the median differences of *R*_*50%*_ and *D*_*2cm*_ between MHE and HE plans were lower than 0.3 and 0.9%, respectively.

For *D*_*max*_, *D*_*2%*_ and the mean dose (*D*_*mean*_) of all rings (excluding *D*_*max*_ and *D*_*2%*_ of Ring1), the median dose reductions ranged from 0.80 to 8.46 Gy (HE plans *vs*. HO plans) and 0.71 to 6.60 Gy (MHE plans *vs*. HO plans), while the value was only 0.17 to 1.20 Gy (HE plans *vs*. MHE plans). The *D*_*max*_ of Ring1 in MHE and HE plans were higher than those in HO plans, where the median differences were 2.66 and 4.30 Gy. No matter *D*_*max*_, *D*_*2%*_ or *D*_*mean*_, the maximum differences were both in Ring2 or Ring3, then the differences got smaller from Ring2 or Ring3 to Ring10. Furthermore, the median *D*_*mean*_ and the median *D*_*mean*_ reductions in both MHE and HE plans *vs*. HO plans were negatively correlated with the distance from tumor to ring (*ρ* ranged from − 0.964 to − 1.000, *P* < 0.001).

## Discussion

The goal of this study is to characterize the relationship between the OARs sparing, dose falloff, and dose heterogeneity level of the target. To our knowledge, this is the first time that, based on an automatic planning program, three heterogeneity levels of target doses in lung SBRT plans have been investigated. We hope that the automatic planning program could make our results more objective and more stable. Moreover, the adjustment precision level of the automatic plan (2 cGy) was more accurate than that of the manual plan (more than 50 cGy).

Unlike conventional radiotherapy, the PTV in SBRT plans mainly involves tumor tissue with its necessary margins arising from tumor motion. Accordingly, it is not necessary for SBRT plans to obtain a higher level of target dose homogeneity [30]. The advantage of the heterogeneous target dose has been demonstrated in the SBRT plans to abdominal malignancies [31]. In the study of Widder et al. on the prescription strategy of SBRT for lung tumors [30], they found that the lower the percentage of the prescription isodose level to the isocenter dose, the faster the dose falloff would be, which was beneficial to improve the normal tissue dose sparing. Our study indirectly confirmed their findings. We found that PTV conformity, OARs sparing, and dose falloff beyond targets improved significantly in MHE and HE plans compared to those in HO plans, while dose improvements in MHE and HE plans were very similar. In addition, the dose sparing of rib and rings derived from the heterogeneous target were associated with the distance from the tumor to normal tissue.

Most planning studies of SBRT have previously been based on manual plans [23–26]. However, the quality of manual IMRT plans has often been dependent on the experience of each planner [22], as well as on the clinical preferences of planners and centers [23–25]. In the present study, all SBRT plans were based on the automated planning software, which guaranteed that the prescription and optimization strategies for each plan were identical. These measures could thereby avoid or minimize the impact caused by the experience or clinical preferences of different planners or centers and guarantee unbiased dose comparison.

At the cost of homogeneity, the *CIs* calculated from MHE and HE plans were superior to those calculated from HO plans, which is inconsistent with Miao et al., who performed a study for the conventional fraction radiotherapy of NSCLC and found that there was no significant difference in *CI* between inhomogeneous and homogeneous plans [19]. A possible explanation for this might be that the SBRT technique pays more attention to conformity than the conventional fraction radiotherapy technique. In the present study, ten rings outside the PTV were used to limit dose to normal tissue and improve PTV conformity. These results suggested that target conformity of SBRT plans could be improved by loosening or abandoning dose homogeneity. Other possible reasons were the PTV volumes in their study was much bigger than those in ours and they also used a Step-and-Shoot technique, not VMAT. In the study by Widder et al. [30], the dose was prescribed to include the PTV with the prescription isodose level specified in a range between 50 and 80% of the isocenter dose. They found that poor CIs was showed in homogeneous plans, not in inhomogeneous plans. Their results were consistent with ours, suggesting that it is not necessary for SBRT plans to obtain a higher level of target dose homogeneity.

Radiation-induced lung toxicity (RILT) is one of the major factors limiting the maximal radiation dose that can be safely delivered to thoracic tumors [32, 33]. Dosimetric parameters are associated with the development of RILT [8]. Chang et al. demonstrated that *MLD* > 6 Gy, *V*_*20*_ > 12%, and ipsilateral *V*_*30*_ > 15% were significantly associated with grade 2–3 radiation pneumonitis (RP) [8]. In our study, both *MLD* and *V*_*20*_ in MHE and HE plans were lower than those in HO plans, as well as the number of patients whose *MLD* and *V*_*20*_ exceeded 6 Gy and 12%, which could be beneficial to reducing the risk of RILT. However, the improvement of lung doses in our study was less than those of Miao et al. who found that mean lung volume received 0–60 Gy and *MLD* was reduced by up to 5.5% (approximately 1.4 Gy). This result may be attributed to fewer limitations on dose falloff in conventional fraction radiotherapy than in SBRT. In their study, only two rings were used to limit dose outside the target. Nevertheless, we still believe lung dose and the risk of RILT can be reduced by loosening or abandoning the dose homogeneity of the target.

Chest wall toxicities are usually associated with peripheral lung tumor treated with SBRT [9–11]. A recent pooled analysis indicated that dosimetric parameters including *D*_*max*_ of 0.5–5 cc and *V*_*30*_ for chest wall or ribs were significantly associated with chest wall pain and rib fracture [9]. Various maximum cutoff doses ranging from 21 to 60 Gy in 2–5 fractions with 0% to 55.7% associated risk of chest wall toxicity were reported [10, 11, 34]. In the present study, the results showed that median rib *D*_*2cc*_ in MHE and HE plans were reduced by approximately 4 Gy compared to those in HO plans, and the number of patients with *D*_*2cc*_ above 30.8 Gy in MHE and HE plans was 3 and 4 less than those in HO plans. Similarly, the median rib *V*_*30*_ was reduced by 2.2%. In addition, the dose improvements of *V*_*30*_ in MHE and HE plans were associated with the distance from tumor to rib. These results support the previous finding that tumor-to-chest wall distance was significantly associated with chest wall pain and rib fracture [9]. For skin, the dosimetric parameters related to skin toxicity included skin *D*_*max*_ [12] and chest wall *V*_*30*_ [35]. In our study, the median dose reduction of skin *D*_*max*_ in MHE and HE plans was approximately 2 Gy compared to HO plans. Therefore, inhomogeneous plans could be beneficial to reducing the risk of chest wall and skin toxicities.

For mediastinal OARs, some dose–response models developed by different SBRT teams show that the probability of radiotherapy toxicities increases with dose, such as *D*_*max*_ of esophagus [13], great vessels [14], trachea, and bronchus [15]. In our study, the median dose of *D*_*2%*_ or *D*_*max*_ reductions of esophagus, great vessels, trachea, and bronchus were less than 2 Gy, which were lower than those for rib. This discrepancy may be due to the fact that peripheral lung tumors are farther from mediastinal OARs than from ribs. Nevertheless, the results still indicated the dose of mediastinal OARs can be reduced by loosening or abandoning homogeneity, which may help reduce the risk of radiation-induced toxicities. Especially for patients treated with radiotherapy combined with chemotherapy or immunotherapy, which has been found to increase the risk of toxicity [36–38].

Steep dose falloff gradients beyond the target are very critical for SBRT, because tissues exposed to high fraction doses are prone to significant dysfunction [7]. Additionally, normal tissue dose tolerances in the context of SBRT are still evolving and only have limited experimentation. For 3D-CRT, beam penumbra at the target edge can be set manually to produce a steep dose falloff outside the PTV with small to no margins. Unlike 3D-CRT, the aperture in VMAT plans was always optimized automatically. Because of this, we used ten rings outside the PTV in this study to limit the dose around the target. We found that *D*_*max*_ and *D*_*mean*_ for all rings (excluding the *D*_*max*_ of Ring1) in MHE and HE plans were reduced by 0.71–8.46 Gy compared to those in HO plans. Analogously, *D*_*2cm*_, *R*_*50%*_, and *R*_*100%*_ in MHE and HE plans were superior to those in HO plans. Therefore, we believe that a steeper dose gradient can be obtained by loosening or abandoning homogeneity of target dose. In addition, the median *D*_*mean*_ improvements of inhomogeneous plans were negatively correlated with the distance from tumor to rings. Combining the results of the ribs and rings, we speculated that normal tissue dose sparing derived from the heterogeneous target could decrease as the distance from the tumor increases.

Comparing MHE and HE plans, there was little to no difference in dose parameters for all OARs or in dose falloff, although different levels of heterogeneity for them were used. A possible explanation for this might be that too flat of a dose curve for PTV in the HE plans results in OARs doses raised by the prescription dose normalization. When *D*_*95%*_ and *D*_*99%*_ were fulfilled by a normalizing prescription dose, a flatter PTV dose line on the DVH led to a dose increase of OARs and other normal tissue, which offset dose sparing obtained by abandoning homogeneity. Therefore, we speculate that 90–125% of the prescription dose may be a tradeoff of heterogeneity levels between target coverage and OARs sparing. There seems to be no further advantage of dose sparing for higher *D*_*max*_.

Several limitations should be addressed here. Firstly, this is a single center study and the sample size is relatively small. Secondly, we have not validated clinical results of different heterogeneity plans. A larger cohort and multi-center study may be necessary to identify dose and clinical outcome differences among SBRT plans with different levels of heterogeneity.

## Conclusions

For SBRT plans of peripheral lung tumor, the conformity of the target, the dose of OARs, and the dose falloff around the target could be improved by loosening or abandoning dose homogeneity. While there was little further dose benefit for a maximum target dose above 125% of the prescription. In addition, the dose sparing of normal tissue derived from a heterogeneous target tended to decrease as distance from the tumor increased.

## Supplementary Information

**Additional file 1: Figure 1.** Total score of three kinds of plan. Horizontal bars indicate median values. HO, MHE, and HE plans = homogeneous, moderate heterogeneous, and heterogeneous plans.

**Additional file 2: Table 1.** PTV dose, monitor units, and statistical comparisons. **Table 2.** OARs dose and statistical comparisons. **Table 3.** Dose parameters of dose falloff beyond PTV and statistical comparisons.

## Data Availability

The datasets generated and/or analyzed during the current study are not publicly available because it contains personal information, but are available from the corresponding author on reasonable request.

## Abbreviations

SBRT: Stereotactic body radiation therapy
VMAT: Volumetric-modulated arc therapy
HO, MHE, and HE: Homogeneous, moderate heterogeneous, and heterogeneous
MLD: Mean lung dose
PTV: Planning target volume
*D*_*max*_: Maximum dose
OARs: Organs at risk
NSCLC: Non-small cell lung cancer
HI: Heterogeneity index
CI: Conformity index
*R*_*50%*_ and * R*_*100%*_: Ratio of 50% and 100% prescription isodose volume to the PTV volume
MUs: Monitor units
*D*_*V*_: The absorbed dose that covers a specified volume *V*
TPBT: Trachea and proximal bronchial tree
*D*_*mean*_: Mean dose
RILT: Radiation-induced lung toxicity
*D*_*min*_: Minimum dose
*D*_*V*_: Absorbed dose that covers a specified fractional volume *V*
*V*_*D*_: Volume that receives at least the absorbed dose *D* Gy
*D*_*2cm*_: Maximum dose (in % of dose prescribed) 2 cm from PTV in any direction
*D*_*2cc*_: Minimuhcfy-anchorm absorbed dose that covers 2 cc of the volumhcfy-anchore
