# Supplementary material for: Influence of target dose heterogeneity on dose sparing of normal tissue in peripheral lung tumor stereotactic body radiation therapy
Source: Radiat Oncol. 2021 Aug 30;16:167. doi: 10.1186/s13014-021-01891-6 (PMC8404286; doi:10.1186/s13014-021-01891-6)
Supplement: Supplementary file 2 — Additional file 2: Table 1. PTV dose, monitor units, and statistical comparisons. Table 2. OARs dose and statistical comparisons. Table 3. Dose parameters of dose falloff beyond PTV and statistical comparisons. [file 13014_2021_1891_MOESM2_ESM.docx]

| **Supplementary Table 1.** PTV dose, monitor units and statistical comparisons. | | | | | | | | |
| --- | --- | --- | --- | --- | --- | --- | --- | --- |
|  | HO plan | MHE plan | HE plan |  | HO, MHE and HE plans | HO and MHE plans | HO and HE plans | MHE and HE plans |
|  | Median (Range) | Median (Range) | Median (Range) |  | *P* ^†^ | *P* ^‡^ | *P* ^‡^ | *P* ^‡^ |
| PTV_*D_max_* (Gy) | 52.29 (49.39-52.80) | 59.37 (58.29-59.98) | 66.38 (59.89-77.12) |  | <0.001 | <0.001 | <0.001 | <0.001 |
| PTV_*D_1%_* (Gy) | 52.23 (49.25-52.72) | 59.11 (56.72-59.82) | 65.31 (59.53-75.21) |  | <0.001 | <0.001 | <0.001 | <0.001 |
| PTV_*D_2%_* (Gy) | 52.11 (49.22-52.62) | 58.89 (55.68-59.71) | 64.02 (59.42-74.44) |  | <0.001 | <0.001 | <0.001 | <0.001 |
| PTV_*D_50%_* (Gy) | 50.30 (48.91-51.20) | 53.39 (50.78-55.39) | 55.32 (53.27-59.75) |  | <0.001 | <0.001 | <0.001 | <0.001 |
| PTV_*D_95%_* (Gy) | 48.00 (47.98-48.18) | 48.00 (47.82-48.28) | 48.00 (47.70-48.44) |  | 0.273 | 0.106 | 0.262 | 0.428 |
| PTV_*D_98%_* (Gy) | 47.31 (46.99-47.82) | 46.91 (46.42-47.37) | 46.60 (45.99-47.35) |  | <0.001 | <0.001 | <0.001 | <0.001 |
| PTV_*D_99%_* (Gy) | 46.83 (46.27-47.71) | 46.04 (45.22-47.05) | 45.75 (44.90-46.69) |  | <0.001 | <0.001 | <0.001 | <0.001 |
| PTV_*D_min_* (Gy) | 44.77 (41.18-47.46) | 42.19 (37.41-45.14) | 41.21 (36.51-44.14) |  | <0.001 | <0.001 | <0.001 | <0.001 |
| PTV_*HI* | 0.095 (0.030-0.106) | 0.224 (0.166-0.242) | 0.321 (0.234-0.477) |  | <0.001 | <0.001 | <0.001 | <0.001 |
| PTV_*CI* | 0.760 (0.438-0.886) | 0.848 (0.739-0.915) | 0.853 (0.711-0.917) |  | <0.001 | <0.001 | <0.001 | 0.165 |
| PTV_MU (MU) | 2029 (1761-2507) | 2282 (2020-3135) | 2504 (2072-3186) |  | <0.001 | <0.001 | <0.001 | <0.001 |
| *Abbreviations:* PTV = planning target volume; *HI* = heterogeneity index; *CI* = conformity index; *MU* = monitor units; *D_max_* = maximum dose; *D_min_* = minimum dose; *D_V_* = absorbed dose that covers a specified fractional volume *V*; HO, MHE and HE plans = homogeneous, moderate heterogeneous and heterogeneous plans. | | | | | | | | |
| ^†^ Friedman test; *P*<0.05 (2-tailed) was considered statistically significant. | | | | | | | | |
| ^‡^ Wilcoxon signed ranks test; *P*<0.017 (α/3, 2-tailed) was considered statistically significant. | | | | | | | | |

| **Supplementary Table 2.** OARs dose and statistical comparisons. | | | | | | | | |
| --- | --- | --- | --- | --- | --- | --- | --- | --- |
|  | HO plan | MHE plan | HE plan |  | HO, MHE and HE plans | HO and MHE plans | HO and HE plans | MHE and HE plans |
|  | Median (Range) | Median (Range) | Median (Range) |  | *P* ^†^ | *P* ^‡^ | *P* ^‡^ | *P* ^‡^ |
| MLD (Gy) | 3.14 (1.69-7.64) | 2.71 (1.46-7.21) | 2.63 (1.39-7.06) |  | <0.001 | <0.001 | <0.001 | 0.001 |
| Whole Lung_*V_5_* (%) | 17.2 (7.4-38.9) | 13.2 (6.7-35.7) | 12.8 (6.4-37.6) |  | <0.001 | <0.001 | <0.001 | 0.206 |
| Whole Lung_*V_10_* (%) | 8.7 (4.4-23.6) | 7.4 (3-22.7) | 6.9 (2.8-21.9) |  | <0.001 | <0.001 | <0.001 | <0.001 |
| Whole Lung_*V_20_* (%) | 3.2 (1.5-12.8) | 2.4 (0.9-11.1) | 2.3 (0.9-10.6) |  | <0.001 | <0.001 | <0.001 | <0.001 |
|  |  |  |  |  |  |  |  |  |
| Rib_*D_2%_* (Gy) | 38.73 (13.6-51.03) | 33.65 (12.8-54.36) | 33.13 (11.41-54.71) |  | <0.001 | <0.001 | <0.001 | 0.024 |
| Rib_*D_1cc_* (Gy) | 40.53 (13.33-51.60) | 35.09 (12.14-56.43) | 33.76 (9.76-57.24) |  | 0.001 | 0.001 | 0.001 | 0.136 |
| Rib_*D_2cc_* (Gy) | 37.71 (13.09-50.93) | 31.25 (7.28-54.10) | 30.83 (4.53-54.42) |  | <0.001 | <0.001 | <0.001 | 0.017 |
| Rib_*V_30_* (%) | 7.2 (0.0-22.7) | 3.6 (0.0-17.3) | 3.4 (0.0-17.2) |  | <0.001 | <0.001 | <0.001 | 0.003 |
| Skin_*D_2%_* (Gy) | 7.93 (5.51-22.1) | 7.22 (4.2-20.25) | 6.8 (4.11-20.24) |  | <0.001 | <0.001 | <0.001 | <0.001 |
| Skin_*D_max_* (Gy) | 20.62 (10.97-52.32) | 17.42 (9.54-59.84) | 17.75 (8.72-62.74) |  | <0.001 | <0.001 | <0.001 | 0.057 |
|  |  |  |  |  |  |  |  |  |
| Spinal Cord_*D_2%_* (Gy) | 7.85 (2.36-19.71) | 6.58 (3.66-16.66) | 6.79 (3.72-16.85) |  | <0.001 | <0.001 | <0.001 | 0.116 |
| Spinal Cord_*D_max_* (Gy) | 8.81 (2.91-24.4) | 7.30 (4.34-20.54) | 7.35 (4.14-20.60) |  | <0.001 | <0.001 | <0.001 | 0.517 |
| Esophagus_*D_2%_* (Gy) | 8.79 (5-16.65) | 7.83 (3.11-14.94) | 8.4 (4.44-16.45) |  | 0.003 | <0.001 | 0.147 | 0.022 |
| Esophagus_*D_max_* (Gy) | 9.95 (5.57-19.12) | 8.81 (3.77-17.04) | 9.47 (4.97-17.66) |  | 0.001 | <0.001 | 0.086 | 0.057 |
| Heart_*D_2%_* (Gy) | 11.07 (1.09-25.69) | 9.89 (0.96-24.33) | 9.69 (1.19-24.67) |  | <0.001 | <0.001 | <0.001 | 0.339 |
| Heart_*D_max_* (Gy) | 15.77 (9.29-50.39) | 14.11 (8.05-51.96) | 14.04 (7.97-52.44) |  | <0.001 | <0.001 | <0.001 | 0.813 |
| Great Vessels_*D_2%_* (Gy) | 12.51 (4.4-25.33) | 10.37 (2.28-20.64) | 10.67 (3.96-20.61) |  | <0.001 | <0.001 | <0.001 | 0.959 |
| Great Vessels_*D_max_* (Gy) | 14.94 (4.87-43.78) | 12.12 (3.52-38.46) | 12.89 (4.73-37.87) |  | <0.001 | <0.001 | <0.001 | 0.829 |
| TPBT_*D_2%_* (Gy) | 10.6 (0.06-21.99) | 8.96 (0.06-18.85) | 9.21 (0.05-20.37) |  | <0.001 | <0.001 | 0.002 | 0.213 |
| TPBT_*D_max_* (Gy) | 13.87 (0.07-32.83) | 12.10 (0.06-26.01) | 11.96 (0.06-27.93) |  | <0.001 | <0.001 | <0.001 | 0.719 |
| *Abbreviations:* OARs = organs at risk; TPBT = trachea and proximal bronchial tree; *MLD* = mean lung dose; *D_max_* = maximum dose; *D_V_* = absorbed dose that covers a specified fractional volume *V*; *D_1cc_* = minimum absorbed dose that covers 1 cc of the volume; *D_2cc_* = minimum absorbed dose that covers 2 cc of the volume; *V_D_* = volume that receives at least the absorbed dose *D* Gy; HO, MHE and HE plans = homogeneous, moderate heterogeneous and heterogeneous plans. | | | | | | | | |
| ^†^ Friedman test; *P* < 0.05 (2-tailed) was considered statistically significant. | | | | | | | | |
| ^‡^ Wilcoxon signed ranks test; *P* < 0.017 (α/3, 2-tailed) was considered statistically significant. | | | | | | | | |

| **Supplementary Table 3.** Dose parameters of dose falloff beyond PTV and statistical comparisons. | | | | | | | | |
| --- | --- | --- | --- | --- | --- | --- | --- | --- |
|  | HO plan | MHE plan | HE plan |  | HO, MHE and HE plans | HO and MHE plans | HO and HE plans | MHE and HE plans |
|  | Median (Range) | Median (Range) | Median (Range) |  | *P* ^†^ | *P* ^‡^ | *P* ^‡^ | *P* ^‡^ |
| Ring1_*D_max_* (Gy) | 51.03 (49.27-53.47) | 53.72 (51.70-55.83) | 55.03 (52.45-60.17) |  | <0.001 | <0.001 | <0.001 | <0.001 |
| Ring1_*D_2%_* (Gy) | 49.74 (48.47-50.99) | 50.00 (48.16-51.94) | 50.47 (47.56-52.01) |  | <0.001 | 0.039 | 0.001 | <0.001 |
| Ring1_*D_mean_* (Gy) | 44.89 (42.45-47.34) | 41.14 (37.99-42.79) | 40.06 (32.34-41.81) |  | <0.001 | <0.001 | <0.001 | <0.001 |
| Ring2_*D_max_* (Gy) | 48.15 (45.49-53.47) | 44.51 (38.37-48.56) | 43.41 (35.32-47.64) |  | <0.001 | <0.001 | <0.001 | <0.001 |
| Ring2_*D_2%_* (Gy) | 44.57 (40.34-48.94) | 38.80 (33.49-42.36) | 37.86 (30.12-41.16) |  | <0.001 | <0.001 | <0.001 | <0.001 |
| Ring2_*D_mean_* (Gy) | 33.69 (28.19-41.29) | 28.03 (22.02-30.24) | 26.40 (16.94-28.81) |  | <0.001 | <0.001 | <0.001 | <0.001 |
| Ring3_*D_max_* (Gy) | 40.93 (34.95-48.15) | 34.62 (28.74-39.89) | 33.85 (26.86-39.42) |  | <0.001 | <0.001 | <0.001 | 0.007 |
| Ring3_*D_2%_* (Gy) | 35.69 (30.26-43.34) | 30.50 (24.78-34.87) | 29.21 (22.79-35.24) |  | <0.001 | <0.001 | <0.001 | 0.001 |
| Ring3_*D_mean_* (Gy) | 23.19 (18.07-31.10) | 19.17 (13.44-21.82) | 18.05 (10.52-21.62) |  | <0.001 | <0.001 | <0.001 | <0.001 |
| Ring4_*D_max_* (Gy) | 33.83 (27.61-41.69) | 28.16 (23.69-34.64) | 27.49 (22.24-33.89) |  | <0.001 | <0.001 | <0.001 | 0.001 |
| Ring4_*D_2%_* (Gy) | 29.35 (24.69-36.32) | 25.13 (20.43-30.12) | 24.16 (18.77-31.02) |  | <0.001 | <0.001 | <0.001 | 0.002 |
| Ring4_*D_mean_* (Gy) | 16.82 (12.62-22.11) | 13.82 (9.36-17.03) | 13.05 (7.42-16.89) |  | <0.001 | <0.001 | <0.001 | <0.001 |
| Ring5_*D_max_* (Gy) | 28.58 (23.49-37.34) | 23.96 (19.98-29.78) | 23.31 (18.52-30.39) |  | <0.001 | <0.001 | <0.001 | 0.003 |
| Ring5_*D_2%_* (Gy) | 24.93 (20.63-32.30) | 21.55 (17.21-26.67) | 20.80 (15.87-27.70) |  | <0.001 | <0.001 | <0.001 | 0.002 |
| Ring5_*D_mean_* (Gy) | 12.69 (9.00-17.28) | 10.53 (6.71-13.92) | 9.87 (5.50-13.82) |  | <0.001 | <0.001 | <0.001 | <0.001 |
| Ring6_*D_max_* (Gy) | 24.61 (20.15-33.33) | 21.09 (17.45-26.90) | 20.42 (15.88-27.19) |  | <0.001 | <0.001 | <0.001 | 0.001 |
| Ring6_*D_2%_* (Gy) | 21.77 (17.99-29.21) | 18.98 (14.60-24.14) | 18.13 (13.54-24.91) |  | <0.001 | <0.001 | <0.001 | 0.002 |
| Ring6_*D_mean_* (Gy) | 10.25 (6.49-13.89) | 8.19 (4.79-11.30) | 7.73 (4.07-11.22) |  | <0.001 | <0.001 | <0.001 | <0.001 |
| Ring7_*D_max_* (Gy) | 21.81 (17.73-29.94) | 18.92 (14.53-24.50) | 18.05 (13.75-24.62) |  | <0.001 | <0.001 | <0.001 | 0.006 |
| Ring7_*D_2%_* (Gy) | 19.50 (16.06-26.67) | 17.00 (12.65-22.04) | 16.12 (11.85-22.53) |  | <0.001 | <0.001 | <0.001 | 0.001 |
| Ring7_*D_mean_* (Gy) | 8.17 (5.04-11.48) | 6.73 (3.71-9.41) | 6.23 (3.06-9.34) |  | <0.001 | <0.001 | <0.001 | 0.000 |
| Ring8_*D_max_* (Gy) | 19.72 (15.80-27.54) | 17.18 (12.54-22.85) | 16.13 (11.81-22.85) |  | <0.001 | <0.001 | <0.001 | 0.005 |
| Ring8_*D_2%_* (Gy) | 17.72 (14.53-24.39) | 15.43 (11.27-20.25) | 14.56 (10.63-20.73) |  | <0.001 | <0.001 | <0.001 | 0.001 |
| Ring8_*D_mean_* (Gy) | 6.66 (4.00-9.41) | 5.58 (2.92-7.76) | 5.12 (2.34-7.70) |  | <0.001 | <0.001 | <0.001 | <0.001 |
| Ring9_*D_max_* (Gy) | 18.15 (14.51-25.25) | 15.69 (11.33-20.99) | 14.69 (10.70-21.20) |  | <0.001 | <0.001 | <0.001 | 0.002 |
| Ring9_*D_2%_* (Gy) | 16.37 (13.06-22.38) | 14.02 (10.15-18.67) | 13.34 (9.62-19.24) |  | <0.001 | <0.001 | <0.001 | 0.001 |
| Ring9_*D_mean_* (Gy) | 5.63 (3.29-7.94) | 4.55 (2.34-6.58) | 4.20 (1.89-6.53) |  | <0.001 | <0.001 | <0.001 | <0.001 |
| Ring10_*D_max_* (Gy) | 16.72 (13.36-23.10) | 14.37 (10.44-19.16) | 13.56 (9.68-19.65) |  | <0.001 | <0.001 | <0.001 | 0.001 |
| Ring10_*D_2%_* (Gy) | 15.10 (11.92-20.61) | 12.93 (9.27-17.32) | 12.34 (8.76-18.06) |  | <0.001 | <0.001 | <0.001 | 0.001 |
| Ring10_*D_mean_* (Gy) | 4.73 (2.73-6.65) | 3.76 (1.95-5.53) | 3.59 (1.58-5.50) |  | <0.001 | <0.001 | <0.001 | <0.001 |
|  |  |  |  |  |  |  |  |  |
| PTV_*R_50%_* | 6.9 (4.4-20.2) | 5 (3.5-10.4) | 4.6 (3.5-8.0) |  | <0.001 | <0.001 | <0.001 | <0.001 |
| PTV_*R_100%_* | 1.2 (1.0-2.1) | 1.1 (1.0-1.2) | 1.1 (1.0-1.3) |  | <0.001 | <0.001 | <0.001 | 0.229 |
| PTV_*D_2cm_* (%) | 59.5 (48.9-77.8) | 49.9 (41.6-62.0) | 48.6 (38.6-63.3) |  | <0.001 | <0.001 | <0.001 | 0.003 |
| *Abbreviations:* PTV = planning target volume; *D_max_* = maximum dose; *D_mean_* = mean dose; *R_50%_* and *R_100%_* = ratio of 50% and 100% prescription isodose volume to the PTV volume; *D_2cm_* = maximum dose (in % of dose prescribed) 2 cm from PTV in any direction; HO, MHE and HE plans = homogeneous, moderate heterogeneous and heterogeneous plans. | | | | | | | | |
| ^†^ Friedman test; *P* < 0.05 (2-tailed) was considered statistically significant. | | | | | | | | |
| ^‡^ Wilcoxon signed ranks test; *P* < 0.017 (α/3, 2-tailed) was considered statistically significant. | | | | | | | | |
